# Cryo-soft X-ray tomography: a journey into the world of the native-state cell

**DOI:** 10.1007/s00709-013-0583-y

**Published:** 2013-11-22

**Authors:** Raffaella Carzaniga, Marie-Charlotte Domart, Lucy M. Collinson, Elizabeth Duke

**Affiliations:** 1 Electron Microscopy Unit, London Research Institute, Cancer Research UK, London, WC2A 3LY UK; 2 Diamond Light Source, Harwell Science and Innovation Campus, Didcot, Oxfordshire OX11 0DE UK

**Keywords:** Cryo-soft X-ray tomography, Cryo-fluorescence, Native state, Correlative, Cells

## Abstract

One of the ultimate aims of imaging in biology is to achieve molecular localisation in the context of the structure of cells in their native state. Here, we review the current state of the art in cryo-soft X-ray tomography (cryo-SXT), which is the only imaging modality that can provide nanoscale 3D information from cryo-preserved, unstained, whole cells thicker than 1 μm. Correlative cryo-fluorescence and cryo-SXT adds functional information to structure, enabling studies of cellular events that cannot be captured using light, electron or X-ray microscopes alone.

## Introduction

**Fig. 1:**
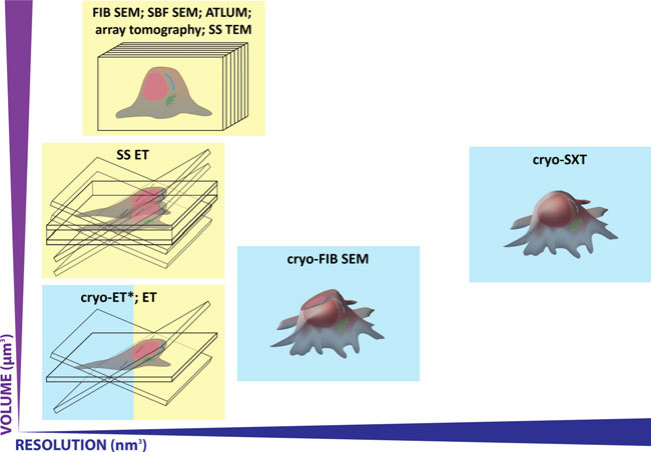
Cryo-SXT inhabits a unique position within the nanoscale 3D imaging landscape. Cryo-electron tomography (*cryo-ET*) can achieve high-resolution imaging of vitreous sections, FIB-milled wafers and thin regions of cells up to a depth of 1 μm. Cryo-SXT extends imaging capability to whole cells up to 10 μm in depth, allowing capture and analysis of complex organelles in the perinuclear region. For larger samples including tissues and model organisms, resin embedding and heavy metal staining is still a requirement, but impressive results can be achieved in terms of volume and resolution using electron tomography (*ET*), serial ET, serial section TEM (*SS TEM*), automated tape lathe ultramicrotomy (*ATLUM*), array tomography, focused ion beam SEM and serial block face SEM

Recent years have seen an explosion of electron microscope technologies for high-resolution 3D imaging of biological samples at nanometre resolution (Fig. 1). These can be subdivided by the method of embedding used to stabilise and protect the sample against damage from the imaging beam and microscope vacuum. For samples embedded in resin, well-established transmission electron microscopy (TEM) techniques (electron tomography, serial section TEM) and, more recently, scanning electron microscopy (SEM) techniques [serial block face SEM, focused ion beam (FIB) SEM, array tomography] are delivering powerful information about cells and tissues in relation to their surrounding ultrastructural environment (reviewed recently by Knott and Genoud 2013). Generation of contrast within the specimen is fundamental to allow features of interest to be distinguished from the resin, which is achieved via the addition of heavy metals during sample preparation in order to scatter electrons that would otherwise pass straight through the sample.

Alternatively, samples may be embedded in vitreous ice and imaged in the cryo-TEM (cryo-electron tomography of thin regions of cells, cryo-electron microscopy of vitreous sections or FIB-milled wafers), reviewed recently by Lucic et al. (2013). Sample preparation is minimal, preserving cells as close to the living state as possible, but depth is severely limited (to <1 μm) and contrast is low since it is generated from a weak phase contrast difference between the biological material and the surrounding ice. Thus, multiple acquisitions from the same area of the sample followed by computational averaging or reconstruction are required to identify features above background noise.

**Fig. 2:**
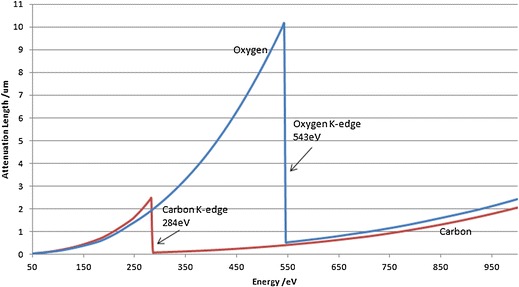
The ‘water window’. Plot of attenuation length (in micrometres) against photon energy (in electron volts) for carbon and oxygen, showing the *k*-absorption edges of the two elements, which define the outer limits of the so-called water window

To date, most 3D EM techniques require the sample to be cut into thin sections due to the limited penetration power of the electron beam. Hard X-rays, with an energy of a few kiloelectron volts to 100 keV, are highly penetrating. However, at these energies, most carbon-rich biological cells and tissues are virtually transparent. Soft X-ray microscopy at a lower energy (∼0.1–1 keV) was developed for use on such samples in the late 1990s (Jacobsen 1999). There is a unique spectral region termed the ‘water window’, bounded by the carbon and oxygen *k*-absorption edges at 284 and 540 eV, respectively, where soft X-rays are strongly absorbed by carbon-rich structures (protein, lipids, membranes) whilst attenuation by surrounding oxygen-dominated material (ice) is minimal (Fig. 2; Wolter 1952). Thus, frozen hydrated biological specimens can be imaged at around 500 eV without the need for additional contrast agents. An additional and significant advantage of imaging biological samples with soft X-rays is that they still have sufficient energy to achieve a penetration depth in the order of 10 μm. As a consequence, soft X-ray microscopes can view most biological cells intact.

Cryo-soft X-ray tomography (cryo-SXT) inhabits a unique position within the biological imaging landscape. It combines the advantages of cryo-techniques (near-native-state imaging, access to atomic composition of the sample, preservation of fluorophores) with advantages of resin embedding (volume imaging). In addition, cryo-SXT is unique in its ability to image intact cells without the need for sectioning. Our recent work (Duke et al. 2013) demonstrates how correlative cryo-fluorescence and cryo-SXT can be used to image cellular processes (the formation of autophagosomes) that are poorly preserved using chemical fixation and resin embedding, but are also too large and complex to be captured within a vitreous cryosection, and reside within the deepest part of the mammalian cell (the perinuclear region), which is inaccessible to cryo-electron tomography. Here, we review the advances in instrumentation and techniques that have opened a new window into the world of the native-state cell.

## Soft X-ray microscopes

Reports of imaging with X-ray microscopes first emerged in the early twentieth century (Goby 1913). However, significant limitations in X-ray sources and optics meant that the technique did not advance significantly for a number of decades. It was not until the 1980s that significant breakthroughs in both X-ray source technology and optics manufacture led to the development of bright, high-intensity beams for the home laboratory. In parallel, huge technological advances in the construction of the first dedicated synchrotron radiation source meant that X-ray microscopy began to move into the mainstream. The development of suitable optics such as grazing incidence mirrors, monochromators, and multilayer optics for energy selection and focusing of the intense X-ray beams, alongside developments in zone plate manufacturing (the optical component in the microscope that governs the overall achievable resolution, assuming a perfect detector and an ideal sample), increased the attraction of X-ray microscopy to instrument developers, technologists and research scientists interested in the ultimate application of the technique. Such was the momentum generated by these advances that a series of international conferences were organised focusing on the latest developments. Conference proceedings from these meetings provide a useful series of historical documents cataloguing progress (Schmahl et al. 1984; Cheng 1987; Shinohara 1990; Michette 1992).

Like any other microscope, regardless of the imaging medium, an X-ray microscope requires a source and a means of delivering the beam to the sample (condenser), collecting the beam once it has passed through the sample (objective) and detecting the resulting image.

Soft X-rays for the microscope can originate from two different sources—plasma sources and synchrotron radiation sources. In a plasma source, the X-rays are generated from a plasma formed from a gas or a liquid with an emission line within the water window (e.g. nitrogen or methanol; Bertilson et al. 2009; Horne et al. 2009). For the synchrotron source, X-rays are produced as a by-product of the relativistic acceleration of electrons around a storage ring; Koch (1991) provides a useful overview. Whilst full-field soft X-ray microscopy does not require photon sources with high brightness, it does need high flux. In addition, there is no requirement for coherence in a full-field microscope, though some systems do operate with partially coherent radiation.

**Fig. 3:**
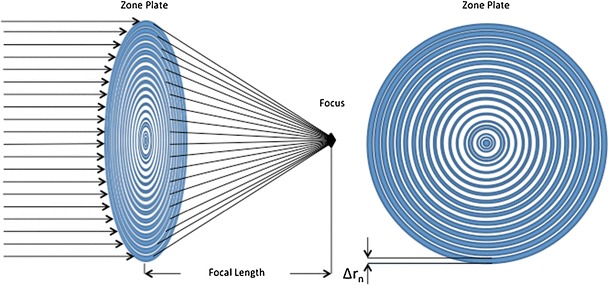
Zone plates for X-ray microscopy. Zone plates are made from concentric rings of material that focus the X-ray beam. The width of the outermost zone (Δ*r*_n_) governs the maximum resolution achievable from data collected using the zone plate

In the transmission X-ray microscope, the X-rays are condensed onto the sample and collected using a diffractive, focusing optical element called a zone plate. Zone plates were invented by Rayleigh (unpublished) and the theory of their operation described by Soret (1875). Decades of research and development in both materials and manufacturing techniques were required to produce zone plates suitable for use with soft X-rays (Baez 1961). Formed from alternating opaque and transparent concentric rings (zones), it is the radius of the outermost ring that determines the resolution of the microscope (Fig. 3). Synchrotron-hosted soft X-ray microscopes routinely operate using two alternative zones plates, one at 40-nm resolution and the other at 25-nm resolution. Unfortunately, zone plates are incredibly inefficient optical elements, with the majority operating at around 10 % efficiency, particularly in the soft X-ray region. Given that the zone plate is fundamental to the operation of the X-ray microscope, it is not surprising that there are worldwide efforts to develop new techniques for zone plate fabrication.

In the early days of soft X-ray transmission microscopes, the fact that zone plates are also chromatically dispersive was employed to good effect in their use as a combined monochromator and condenser. Some modern microscopes still use this arrangement as it provides a cost-effective method of delivering suitable X-rays to the sample. However, an alternative arrangement exists where the desired X-ray energy is selected by a monochromator, which is then focused onto the sample using a capillary condenser.

The current detector of choice for soft X-ray microscopy is a Peltier-cooled, back-illuminated CCD detector. No scintillator is required as the X-rays are detected directly by the CCD detector. The active CCD chip of the detector remains inside the vacuum space of the microscope.

## Three-dimensional imaging with soft X-ray tomography

It is possible to image structural detail within the sample in a 2D projection image taken with the sample perpendicular to the beam (at a 0° tilt). However, high-resolution information from the volume of the sample is only distinguishable using tomography. Soft X-ray tomography (SXT) follows a similar protocol to electron tomography, where images are collected as the sample is tilted in the path of the beam (tilt series) and the data are then computationally reconstructed into a high-resolution volume (tomogram).

The major challenge in SXT data collection is the need to place the objective zone plate millimetres from the sample. However, the available space (focal length) is determined by the resolution of the zone plate in combination with the X-ray energy, which forces a number of geometric and physical constraints on the microscope. Great care needs to be taken in setting up the microscope to ensure that there are no clashes with the sample mount as the sample is rotated during tomographic data collection.

## SXT imaging at cryo temperatures

As with other high-resolution imaging methods, protection against radiation damage is essential to preserve sample integrity during data collection (Sayre et al. 1977; Folkard 1992). Cryo-fixation methods, based on the pioneering work of Dubochet’s team (Dubochet et al. 1988; Dubochet 2012), have been demonstrated to protect biological samples from visible structural damage in cryo-TEM (Glaeser and Taylor 1978). Similarly, the application of high-resolution X-ray imaging to biological samples only became feasible with the development of cryogenically cooled microscopes, with stable cooled sample mounts being the key to collecting high-quality data. This was especially important when moving from 2D to 3D imaging using cryo-SXT (Schneider 1998; Maser et al. 2000; Wang et al. 2000; Weiss et al. 2000), where the sample is exposed to multiple doses during tilt series data collection.

There are currently three full-field cryo-soft X-ray microscopes in routine operation at synchrotrons with a number at various stages of design, commissioning and installation (Table 1). One of the first cryo-SXT beamlines, at the Advanced Light Source (ALS) in Berkeley (California, USA; Meyer-Ilse et al. 2000), developed a custom-designed cryo-stage that could accommodate cells mounted within a quartz capillary. Advances in cryo-TEM techniques and technologies have fed into the design of the sample holders and stages of the more recent cryo-soft X-ray microscopes. The BESSY II microscope (Berlin, Germany) accepts samples supported on high tilt (HZB-2) grids (Hagen et al. 2012) using a modified stage (FEI Company) and cryo-holder (Gatan Inc.) derived from electron tomography. The microscopes at ALBA (Barcelona, Spain) and the Diamond Light Source (Oxford, UK) build on the philosophy and designs developed for the automation of data collection for cryo-TEM. Samples are supported on conventional 3-mm TEM grids, with up to four grids loaded into the microscope at a time. The system, developed by Xradia (now Carl Zeiss X-ray Microscopy), includes an auto-mounter that can pick and place a cartridge containing a grid into the data collection position and then return it to the storage system after imaging. Thus, the microscope can potentially be used for several days (or even weeks) without manual intervention to reload grids and the opportunity exists for the development of remote operation protocols.

**Table 1:** Operational synchrotron-based facilities for cryo-SXT imaging

Facility	Location	Source	Microscope			Sample		Comments
			Type	Atmosphere	Cooling	Mount	Stage	
ALBA	Barcelona, Spain	Synchrotron, Bending magnet	Xradia (now Carl Zeiss X-ray microscopy)	In-vacuum	Nitrogen	3-mm grid	Vertical cryo-stage	Automounter for up to 4 grids
ALS	Berkeley, CA, USA	Synchrotron, Bending magnet	Custom	In-air	Helium	Capillary	Custom vertical cryo-stage	
BESSY II	Berlin, Germany	Synchrotron, Undulator	Custom	In-vacuum	Nitrogen	Custom grid	Horizontal cryo-stage (modified from FEI)	Fluorescence microscope co-located in microscope chamber
Diamond	Didcot, UK	Nitrogen plasma source (due to move to synchrotron bending magnet in 2014)	Xradia (now Carl Zeiss X-ray microscopy)	In-vacuum	Nitrogen	3-mm grid	Vertical cryo-stage	Automounter for up to 4 grids

A major advantage of the capillary method is the ability to image the specimen over a full 360° rotation. For microscopes that use grids, the close proximity of the zone plate means that data collection angles are usually limited to −70° to +70°, but due to the increased sample thickness at high tilt, there is often minimal information in the extreme images. To gather information from high tilts where the sample is thicker, a variable exposure time strategy can be adopted. This increases the exposure time by a factor related to 1/cos (sample angle), so that features remain visible even at the high angles. However, the restricted rotation range leads to a missing wedge of information, which will remain a limitation of these microscopes due to the high-resolution zone plate.

## Sample preparation for cryo-SXT

Vitrification is the only step required to prepare biological samples for cryo-SXT. The purpose of vitrification is to combat the radiation damage effects of the X-rays at doses up to 10^10^ Gy (Schneider et al. 1995) whilst also protecting the sample from the vacuum inside the microscope. Successful vitrification is crucial to obtain the best resolution in cryo-SXT. Three methods of cryo-preservation have been used so far: rapid freezing by a blast of liquid nitrogen-cooled helium gas, plunge freezing and high-pressure freezing. The choice of method is dependent on the sample holder and geometries available on the selected beamline.

For the ALS microscope, capillaries are pulled to a final diameter ideally matching that of the cells to be studied, with the walls of the capillary as thin as possible to minimise X-ray absorption by the quartz. The samples are then pipetted into the capillaries, prior to sealing and freezing (Larabell and Le Gros 2004). Capillaries may be rapidly frozen by a blast of liquid nitrogen-cooled helium gas (Meyer-Ilse et al. 2000) or plunge-frozen in liquid nitrogen or ethane (Weiss et al. 2000). The capillary method is optimal for yeast suspensions and has been extensively described by Larabell’s group (Larabell and Le Gros 2004; Le Gros et al. 2005; Parkinson et al. 2008, 2012; Uchida et al. 2009; McDermott et al. 2012).

**Fig. 4:**
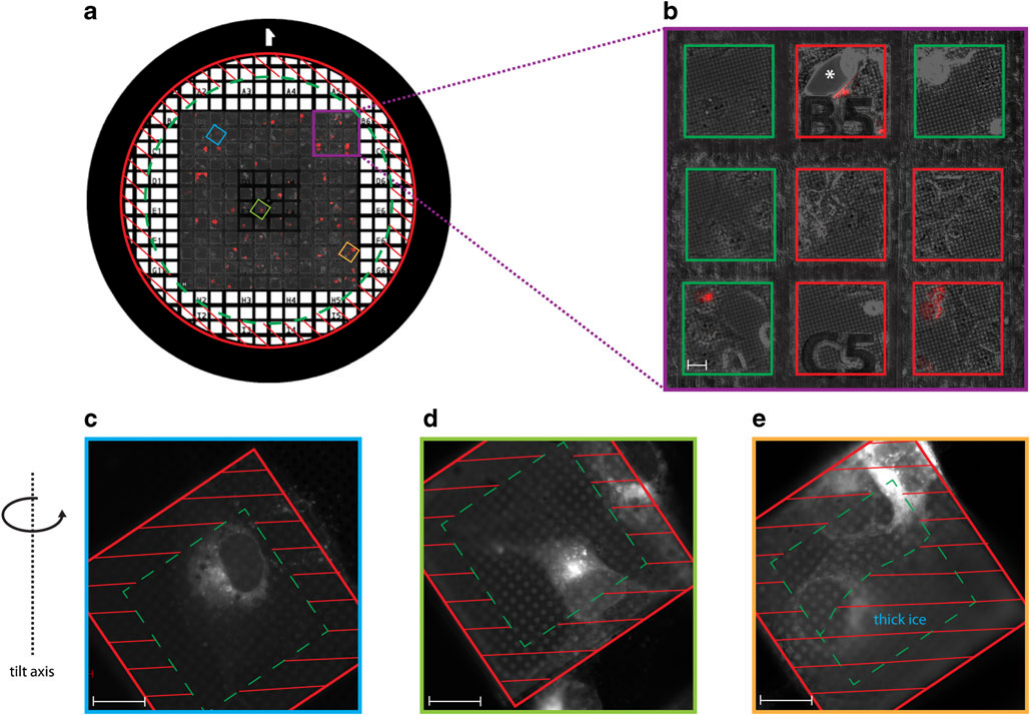
Selecting suitable cells for cryo-SXT data collection. Example shows COS-7 cells grown on a Quantifoil^TM^ R2/2 holey carbon-coated G200F1 (Gilder Grids Ltd.) gold grid and transfected with an mCherry-tagged membrane marker. **a** G200F1 grid map showing the area suitable for data collection (*dashed green line*). Cells too close to the grid rim (*red lines*) are unsuitable for tomography data collection as images will be blocked by the grid at high tilt. Superimposed on the grid map is a confocal tile scan image (one of eight 1.5-μm *z*-sections) from live COS-7 cells transfected with an mCherry-tagged membrane marker, imaged at ×20 magnification with phase contrast and fluorescence channels overlaid. This enables rapid evaluation of grid quality before freezing as well as a pre-selection of cells of interest. **b** Enlargement of region of interest illustrating the pre-selection process. *Red squares* correspond to areas unsuitable for cryo-SXT due to high cell density, absence of cells or a tear in the carbon film (*asterisk*). *Green squares* correspond to potentially good positions (to be validated by cryo-fluorescence imaging). **c–e** Cryo-fluorescence images (after plunge freezing) of three different positions on the same grid [colour-coded positions shown in (**a**)]. The *dashed green line* indicates the area suitable for data collection. Cells too close to the grid bars (**d**, **e**) or in areas where the ice is too thick (**e**) are unsuitable for tomographic data collection (*red lines*). *Scale bars*, 20 μm

The established method for the study of adherent cells involves grids coated with a film of holey carbon, also used in cryo-TEM (Sartori et al. 2007). When using grids, there are several factors that should be considered (Fig. 4). Grid pattern is critical as it is used to relocate individual cells during correlative imaging, but must not interfere with tomographic data collection (Hagen et al. 2012; Duke et al. 2013). In addition, the use of pure gold grids is imperative if cells are to be grown on the support (Agarwal et al. 1989; otherwise, copper grids can be used to support cell suspensions or isolated cell components. The carbon film can be homemade or purchased on grids with different patterns of holes (Hagen et al. 2012; Duke et al. 2013). For cell cultures, grids need to be made hydrophilic by glow discharge before plating the cells. The final cell density is a critical parameter in obtaining good quality results; too high a cell density will lead to overlapping cells and a loss of final resolution. Cell position is also critical, with cells close to the grid rim and grid bars unsuitable for data collection because the image is blocked by the grid at high tilt. The addition of fiducial markers to grids, prior to cell culture and again prior to plunge freezing, is essential for accurate tomographic reconstruction as image alignment (and thus reconstruction quality) is dependent on the number of gold beads in the field of view (Hagen et al. 2012; Duke et al. 2013).

Grids can be plunge-frozen in liquid nitrogen-cooled liquid ethane (Maser et al. 2000; Duke et al. 2013) or a mixture of ethane and propane (Hagen et al. 2012; Cinquin et al. 2013). The use of homemade (Hagen et al. 2012) and commercial vitrification systems (Duke et al. 2013) has been reported. Grid blotting is a critical parameter, determining ice thickness, and must be performed from the opposite side of the grid to the cells. In addition, the use of high-pressure freezing has recently been proposed as an alternative method of sample vitrification for the preparation of thicker samples (20 μm; Weiner et al. 2013).

Once the samples have been frozen, all steps from transfer between microscopes to sample screening must be performed with a view to preserving vitrification and sample integrity.

## Image analysis

To date, the majority of SXT datasets have been processed with software packages that have been designed for the equivalent electron tomography data: IMOD (http://bio3d.colorado.edu/imod), ImageJ (http://rsbweb.nih.giv/ij/index.html) and Bsoft (Heymann 2001; Heymann and Belnap 2007; Heymann et al. 2008). Comparisons have been made of the data output from the different softwares (Hummel et al. 2012) and also of the different algorithms within IMOD (Duke et al. 2013). The reconstruction results are generally impressive, with the caveat that no account is taken of the specifics of the interaction of X-rays with the sample. Some groups are attempting to model the transmission of X-rays through both the microscope and the sample with a view to incorporating this information into algorithms, so that the true nature of the X-ray images can be understood (Oton et al. 2012). A significant challenge is the necessity to account for the depth of focus of the soft X-rays. Despite the fact that biological cells are relatively thin, even when embedded within a layer of vitreous ice (generally <10 μm), it is highly likely that if the top of the cell is in focus then the bottom of the cell is not. Until this work is completed and validated, we continue to use software designed for electron imaging.

**Fig. 5:**
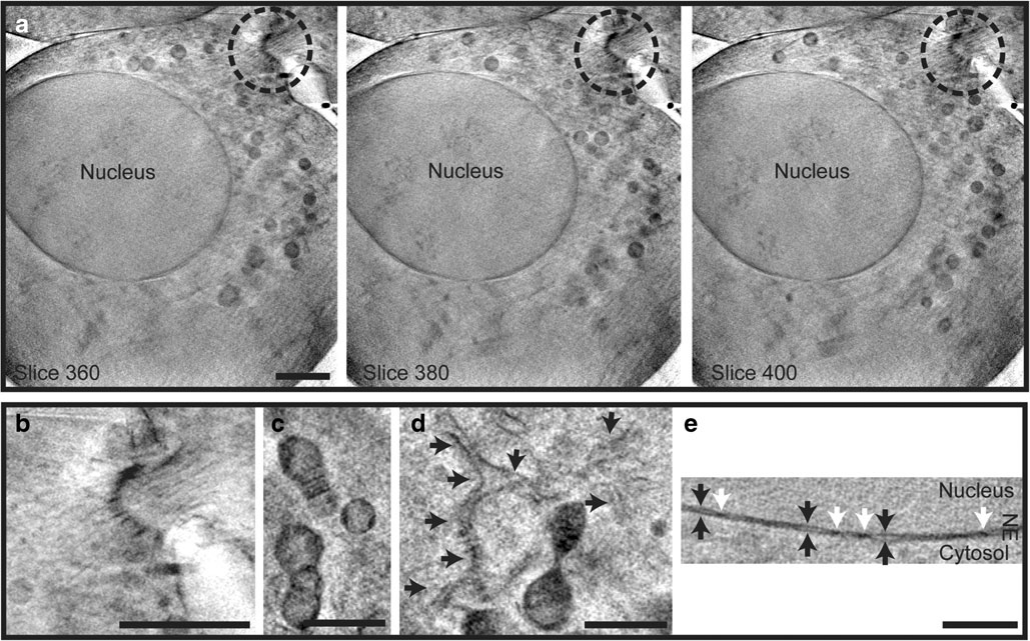
Cryo-SXT of native-state ultrastructure in mammalian cells. **a** Three images taken from a reconstructed tomogram of a dividing G361 cell showing the native-state ultrastructure of the nucleus and an intercellular bridge (*circled*), shown at higher magnification in (**b**). Other structures that can be seen include the mitochondria with christae (**c**), endoplasmic reticulum (*arrows*) (**d**) and the nuclear envelope (**e**) where both bilayers (*black arrows*) and nuclear pores (*white arrows*) can be resolved. Data were collected by M. Razi, L. Collinson and E. Duke with P. Guttmann, S. Werner, K. Henzler and G. Schneider at the BESSY II facility in Berlin, Germany, as described in Duke et al. (2013). *Scale bars*, 2 μm (**a**, **b**) and 1 μm (**c**–**e**)

The resolution of X-ray tomography data has so far been assessed using methods optimised for equivalent EM datasets, namely ‘leave-one-out’ approaches (Cardone et al. 2005). This results in a resolution assessment closely following that predicted by theory. However, structural resolution is more difficult to assess and is affected by multiple parameters including tilt range, sample thickness and vitrification quality. In addition, some features in the data stand out due to their high contrast compared to the surrounding material, including carbon-dense membranes. For example, it is possible to see the space between the two bilayers of the nuclear envelope, a distance of approximately 20 nm (Muller et al. 2012; Duke et al. 2013; Fig. 5), even though the actual resolution of the data does not exceed 40 nm^3^ when using a 40-nm zone plate. Moving forward, it will be very important to establish a method for determining the resolution of the data that neither over- nor underestimates ‘structural’ resolution. Improvements in data quality may be possible as advances are made in sample preparation protocols, microscope technology, data collection and reconstruction strategies, especially if a full model of the X-ray’s interaction with the samples can be developed.

Selection of features of interest within the data for 3D modelling has been reported using both automated and manual techniques. The contrast of biological structures in tomograms collected over a full 360° tilt series is directly related to their X-ray absorption and, thus, their atomic composition. This means that organelles can be automatically identified based on density using the linear absorption coefficient (LAC; Parkinson et al. 2008). However, recent experience suggests that it is more challenging to associate the LAC with specific structures in tomograms reconstructed from partial tilt series of cells grown on grids. This may be due to the missing wedge of information, or perhaps due to the crowded cytoplasm of the mammalian cells. Thus, manual segmentation is required to select specific membrane-bound organelles in data collected from mammalian cells grown on grids (Duke et al. 2013).

## Imaging biological structures using cryo-SXT

Cryo-SXT has been applied to a range of ‘unicellular’ samples that fall within the estimated 10-μm optimal sample thickness limit, from individual vaccinia virus particles (Carrascosa et al. 2009) to whole mammalian cells (Scherfeld et al. 1998; Meyer-Ilse et al. 2001; Chichon et al. 2012; Muller et al. 2012; Hagen et al. 2012; Clowney et al. 2012; Drescher et al. 2013; Duke et al. 2013), with the majority of work to date focused on yeast cells (Larabell and Le Gros 2004; Le Gros et al. 2005; Parkinson et al. 2008; Uchida et al. 2009, 2011; McDermott et al. 2012).

Yeast ultrastructure has historically been difficult to image due to the barrier of the thick cell wall, which resists the penetration of chemical fixatives. Traditionally, the cell wall is removed to make spheroplasts prior to chemical fixation, but this disrupts the native structure of the cells. Fast freezing, either by plunge freezing or high-pressure freezing, has replaced chemical fixation as the method of choice (Giddings et al. 2001). However, cells are then too thick to image in the electron microscope without vitreous sectioning or the addition of stains via freeze substitution followed by resin embedding, serial sectioning and electron tomography. Cryo-SXT has advanced the field through whole-cell analysis at sub-diffraction limit resolution at near-native state. The X-ray ultrastructure of native-state *Saccharomyces cerevisiae* and *Schizosaccharomyces pombe* have been extensively characterised by Larabell’s group, including the size and morphology of the nucleus, mitochondria, vacuole and lipid droplets (Larabell and Le Gros 2004; Gu et al. 2007; Parkinson et al. 2008; Uchida et al. 2011) at a resolution of at least 60 nm (Larabell and Le Gros 2004).

Early work on mammalian cells focused on cytoskeletal elements and the endoplasmic reticulum (ER) in PtK2 cells, either air-dried or plunge-frozen (Scherfeld et al. 1998). More recently, extensive investigation of a mouse adenocarcinoma cell line has seen the beginnings of an atlas of mammalian cell X-ray ultrastructure (Muller et al. 2012), including images of chromatin, the nucleus and nucleolus, nuclear membrane and the perinuclear space, mitochondria, ER, Golgi and filaments (most likely actin bundles and microtubules), to which we contribute here with further images of mammalian cell organelles, including an intercellular bridge in a mitotic cell (Fig. 5). Vaccinia virus has also been imaged in infected cells, where mature particles can be distinguished in the perinuclear region and cell periphery. Mature virus particles are wrapped in multiple membrane layers, hints of which can be seen, but the fine structure cannot be resolved. In plant cell biology, features down to 30 nm can be distinguished in the alga *Chlamydomonas reinhardtii* (Hummel et al. 2012), including nuclear envelope membranes, nucleolus, chloroplasts with thylakoids, mitochondria, lipid bodies, basal apparatus and flagellar microtobule doublets. However, the resolution limit of cryo-SXT, currently estimated at around 30 nm^3^ (Muller et al. 2012), means that the two leaflets of a lipid bilayer remain out of reach.

Organelles that can be recognised by morphology alone in all sample types imaged to date include the nucleus, lipid bodies and mitochondria. However, in our recent work, using a range of cell types, we found that some organelles were not as easy to recognise as first thought, particularly in data collected using a partial tilt series (Duke et al. 2013). Here, the LAC does not correlate to specific organelles, with dense mitochondrial membranes resembling some organelles of the endo-lysosomal system. Thus, there is a need to use immunolabelling to unambiguously assign some organelles in cryo-SXT images. In early work, the microtubule network was labelled in EPH4 cells, and nuclear pore complexes and RNA splicing factors were labelled in human mammary epithelial tumour cells using silver-enhanced immunogold labelling (Meyer-Ilse et al. 2001). Ashcroft et al. (2008) demonstrated the potential of titanium oxide (TiO_2_) nanoparticles as soft X-ray probes using streptavidin-conjugated TiO_2_ labelling biotinylated microtubules. A TiO_2_ absorption edge lies within the water window, so it can be specifically distinguished from cellular matter and also separated from gold labels in the same sample for dual immunolabelling. Most recently, cryo-SXT has been correlated with surface-enhanced Raman scattering of silver nanoparticles in 3T3 and J774 cells (Drescher et al. 2013), and compartments of the endocytic pathway in mammalian cells have been identified using fluid phase uptake of gold-conjugated anti-transferrin receptor antibodies (Duke et al. 2013).

Although the application of cryo-SXT to biological questions is still in its infancy, important information is beginning to emerge. One of the simplest problems that can be answered with cryo-SXT is that of accurate estimation of cell and organelle volumes at a resolution higher than light microscopy and where volume is unaffected by the fixation and dehydration artefacts common to electron microscopy. The effect of antifungal peptides on *Candida albicans* morphology (Uchida et al. 2009) and the volumetric ratios of organelles to cells in various yeast strains (Uchida et al. 2011) have been analysed. In addition, cryo-SXT has been proven valuable for the study of host–pathogen interactions of the malaria parasite *Plasmodium falciparum* within erythrocytes, particularly due to the detection of compositional information from the iron-rich hemozoin crystals deposited as the parasite matures (Hanssen et al. 2011, 2012).

## Moving from structure to function with correlative cryo-fluorescence and cryo-SXT

Functional studies using cryo-SXT have been aided by correlation of fluorescent protein markers with X-ray ultrastructure. This can be done in several ways. Confocal microscopy images can be collected from cells grown on finder grids prior to cryo-fixation, enabling tracking of events within live cells. Grids can also be imaged post-freezing using cryogenic light microscopes, either stand-alone (Duke et al. 2013) or integrated into the beamline (Schneider et al. 2012). The combination of screening on a stand-alone cryostage in the home laboratory (Duke et al. 2013) and relocation of cells of interest using the visible light microscope within the X-ray chamber (Schneider et al. 2012; Hagen et al. 2012; Duke et al. 2013) makes best use of precious beamtime, but with every transfer of the frozen sample, there is the possibility of ice contamination. In addition, for correlative work where efficient transfer of samples between different instruments is required, common sample mounts must be developed that are compatible with existing facilities.

Some of the first applications of correlative light and cryo-SXT used fluorescent markers to identify and quantify vacuoles (Le Gros et al. 2009) and mitochondria in yeast (Parkinson et al. 2008). More recent workflows, using correlative cryo-fluorescence and cryo-SXT (cryo-CLXM) techniques (Le Gros et al. 2009; McDermott et al. 2009; Schneider et al. 2012; Duke et al. 2013), are starting to deliver results close to those achieved by correlative light and electron microscopy, enabling the identification of structures based on fluorescent protein localisation where morphology alone is insufficient. Virus-induced vesicular structures have been identified in the nucleus of cells associated with an expanded nucleoplasmic reticulum (Hagen et al. 2012), and the formation of early autophagosomes from endoplasmic reticulum hot spots has been characterised in mammalian cells (Duke et al. 2013).

## Outlook

As with any scientific investigation, it is vital to ensure that the correct imaging technology is used to answer the question being posed since every technique has associated advantages and drawbacks. Cryo-SXT has enabled us, for the first time, to visualize any region within intact cells at near-native state. It is particularly suited to studies where the subcellular structure under investigation is located within the deepest part of the cell and would be compromised by traditional chemical preparation processes. The power of cryo-SXT is significantly enhanced via cryo-CLXM, where localization of fluorescent proteins within complex organelles can be achieved. Given that the technique is still in its infancy, there are myriad possibilities for developments, particularly focused on improving resolution (currently an order of magnitude lower than EM of cells) and directly detecting the atomic composition of structures. Thus, we expect cryo-SXT to develop as a powerful imaging tool that can contribute greatly to our understanding of cell complexity.
